# Surgical management and outcomes of vesicovaginal fistula: a 12-year experience in a tertiary center

**DOI:** 10.3389/fmed.2026.1757810

**Published:** 2026-05-08

**Authors:** Jianshu Ni, Mengyi Zhang, Xiang Wan, Bin Xu, Haijun Yao

**Affiliations:** 1 Department of Urology, Shanghai Ninth People’s Hospital, Shanghai Jiao Tong University School of Medicine, Shanghai, China; 2 Department of General Surgery, Shanghai Ninth People’s Hospital, Shanghai Jiao Tong University School of Medicine, Shanghai, China

**Keywords:** irradiated, tissue interposition, transvaginal, urinary diversion, vesicovaginal fistula

## Abstract

**Background and objectives:**

Vesicovaginal fistula (VVF) is a devastating condition for female patients. We aimed to summarize our experience in the management of VVF and the surgical outcomes at our center and propose an algorithm for clinical practice.

**Materials and methods:**

We conducted a retrospective study of patients with VVF who underwent surgical treatment at our center between January 2013 and December 2024. All patients were classified into non-irradiated and irradiated groups. Medical data were analyzed, including patient demographics, fistula characteristics, vaginal width, surgical procedures, surgical outcomes, and perioperative complications.

**Results:**

We included 64 patients with VVF (20 non-irradiated and 44 irradiated). The fistula size in the irradiated group was mostly > 1.5 cm, whereas the size was <1.5 cm in the non-irradiated group. The vaginal width was > 3 cm in the fistula repair group, whereas it was < 3 cm in the urinary diversion group. For the non-irradiated group, we all performed fistula repair surgery, while only 47.7% in the irradiated group, where the remaining underwent urinary diversion (29.5%) or fistula repair + urinary diversion (22.7%). The irradiated group had a higher fistula recurrence rate than the non-irradiated group in both transvaginal (18.5% vs. 6.25%) and transabdominal (50% vs. 0%) approaches.

**Conclusion:**

We proposed an algorithm for the surgical management of VVFs based on radiation history, fistula size (>3 cm), and vaginal width (<3 cm). We suggest that transvaginal approach is the first option for fistula repair. For irradiated VVFs, transvaginal fistula repair with tissue interposition combined with permanent cystostomy demonstrated high success in our series, which is a valuable method.

## Introduction

Vesicovaginal fistula (VVF) is an abnormal connection between the urinary bladder and the vagina, causing permanent urine leakage through the vaginal canal (1). VVF is one of the most debilitating and devastating gynecological and obstetric complications (2). It is often caused by childbirth in developing countries, while it is mostly iatrogenic (surgery or radiation) with a relatively low incidence in developed countries (3, 4).

Vesicovaginal fistulas significantly worsen the patients’ quality of life both socially and psychologically, which led to depression, loss of self-esteem, self-worth and identity (5). Effective treatments, especially success fistula repair, are always highly demanded and anticipated. As conservative treatments usually fail to achieve successful fistula healing, surgical treatments, including fistula repair and urinary diversion, are in high demand. Surgical treatments can be performed via vaginal, abdominal, laparoscopic, and robotic approaches. Various factors, including etiology, fistula site, fistula size, and history of radiation, influence the decision of surgical strategy and predict the outcomes for VVF repair (6). In particular, radiation-related VVF is a rare and challenging complication following radiation treatment for pelvic malignancies (7). After radiation therapy, the blood supply of the target decreases, and the wound healing ability is dramatically deteriorated. Abundant evidences demonstrate that fistulas in patients with history of pelvic radiation are more complex to repair and have lower rates of successful repair (8).

As most VVFs require surgical treatment, a thorough preoperative evaluation is required. Timing, surgical methods (approach, technique, flap utilization), and postoperative care must be optimized for successful management. However, no standard guidelines have been established in VVF management algorithms, and no clear recommendations can be made (9). The management of VVF is still individualized and dependent on the surgeon’s experience (10).

This study aimed to summarize our 12-year experience in the management of VVF and to report surgical outcomes with regard to the type of repair, success rate of fistula repair, and complications. We divided the patients into two groups depending on whether they received radiation therapy. Finally, an algorithm was established for clinical practice in VVF treatment.

## Materials and methods

### Study design, setting and patients

We conducted a retrospective study to identify all patients with VVF who underwent elective surgical treatment at our tertiary center from January 2013 to December 2024, with a follow-up period of >6 months. VVF was diagnosed based on a combination of symptoms (vaginal leakage of urine) and findings on radiological examinations, such as retrograde/voiding cystourethrogram, CT urography (CTU), and pelvic MRI. Exclusion criteria comprised: remaining or concomitant malignant tumors; incomplete clinical records; follow-up < 6 months; and severe liver and kidney dysfunction or systemic diseases. All patients were separated into two groups according to whether they received radiotherapy. Medical records were reviewed on patient demographics and characteristics, including comorbidity, etiology, and whether they underwent radiotherapy and chemotherapy.

### Preoperative evaluation

Preoperative evaluation included fistula characteristics, vaginal width, and previous history of fistula repair. Physical vaginal examination was routinely performed to evaluate the fistula site, size, vaginal width, and vaginal flexibility. A duckbill speculum was placed at the vaginal introitus and the maximum width was measured at that level as vaginal width. Cystoscopy and vaginoscopy were further performed to assess the fistula site, size, and the condition of bladder and vagina, especially when the fistula is not well-shown through vagina. Retrograde/voiding cystourethrogram was applied in all cases for bladder morphology. Ultrasound or CT urography were selectively performed when upper tract function assessment is needed.

### Surgical strategy selection and surgical outcomes

The surgical strategy was individually selected by the surgeon according to the anatomical complexity of the fistula (size, Goh classification, location, vaginal width, condition of bladder and vagina, etc.), history of pelvic surgery, history of radiation, and patient willingness. A multidisciplinary fashion was undertaken when colorectal, gynecological, and plastic surgeons were required. Fistula repair is always the first choice unless the fistula is too large or the tissue condition is not capable for repair. Tissue interposition is employed to strengthen the repair in cases with large or recurrent fistulas or poor tissue quality. Urinary diversion is the last option for those with multiple failures or inoperable pelvic condition. Transvaginal or transabdominal approach was determined by the fistula location and vaginal accessibility that if the fistula can be reached after exposure. Surgical procedures (approach, type of surgery, tissue interposition usage) and surgical outcomes (operative time, blood loss, postoperative hospital stay, and fistula recurrence) were all recorded.

### Postoperative follow-up and outcome definition

Postoperatively, the urethral catheter was remained for 4 weeks for patients receiving fistula repair. Besides, antibiotics were routinely used, and anticholinergic medications were administered to control bladder spasms according to certain conditions. Patients were advised to avoid sexual intercourse for 3 months post repair. Overall, patients were followed up postoperatively for at least 6 months. Follow-up visit was scheduled at 1 month, 3 months, 6 months and 12 months. After this, patients were followed yearly. Each visit included clinical history and physical examination. Retrograde/voiding cystourethrogram and cystoscopy were performed at 1 month and 3 months to confirm bladder integrity. Accordingly, successful VVF closure was defined as complete resolution of urinary leakage from vagina and no fistula recurrence at 3 months follow-up.

### Perioperative complications

Perioperative complications were recorded including early complications (urinary tract infection, surgical site infection, bladder spasm, anastomotic leak, postoperative hemorrhage, postoperative ileus) and late complications (overactive bladder, underactive bladder, chronic pelvic pain). The early complications were further graded using the Clavien-Dindo system.

### Statistical analysis

Categorical variables were reported as numbers and percentages (n, %) and analyzed using the chi-squared test and Fisher’s exact test. Continuous variables are presented as mean ± standard deviation (normally distributed) and median with interquartile range (non-normally distributed) and were analyzed using the independent *t*-test and Mann–Whitney *U* test, respectively. *p*-values < 0.05 were considered significant. Statistical analyses were performed using SPSS Version 25.

## Results

**Figure 1:**
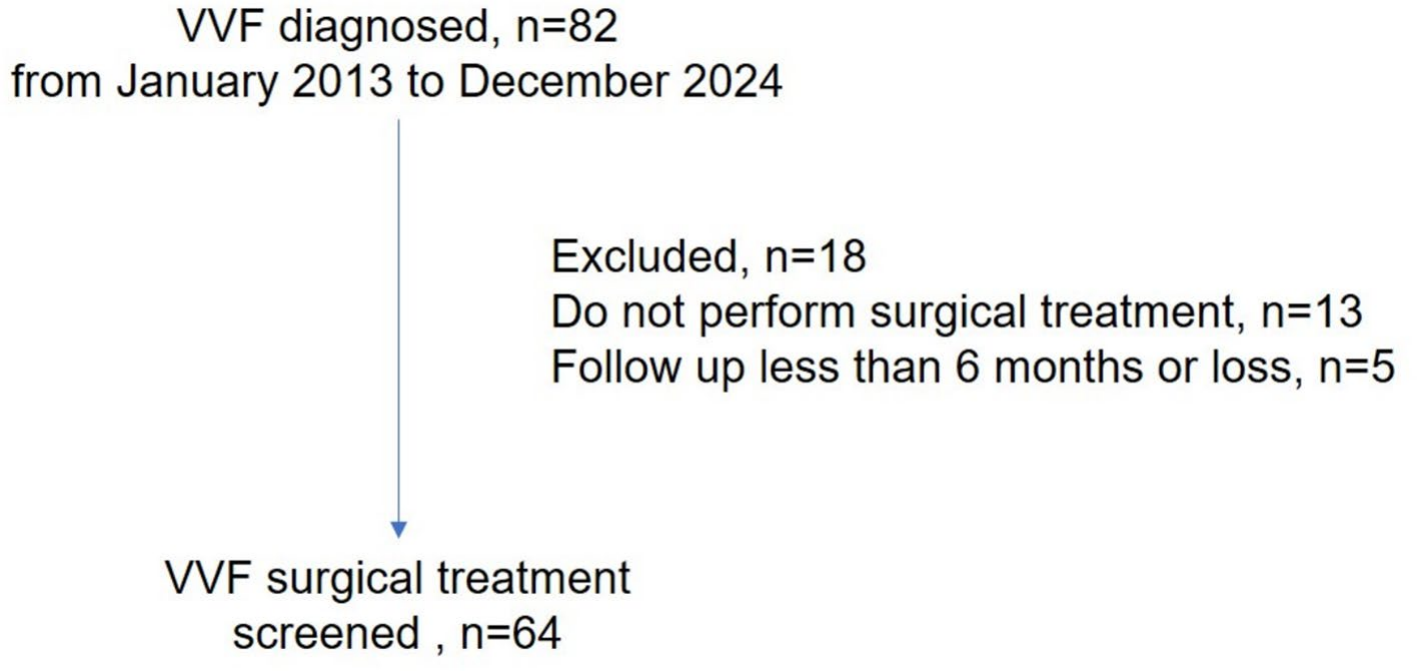
Flowchart of patient’s inclusion.

We identified 64 patients who underwent surgical treatment with a follow-up period of > 6 months at our center (Figure 1). Patient demographics and characteristics are displayed in Table 1. Of note, patients in the irradiated group were significantly older and had a later onset of VVF diagnosis, with a mean age of 54.5 and 50.1, whereas the mean patient age and age at diagnosis were 46.3 and 43.8, respectively, in the non-irradiated group. There were no significant differences in BMI, smoking, or comorbidities between the two groups. The major etiology of VVF in our cohort was malignancy (71.9%). Specifically, the major etiology was benign gynecologic surgery (85%) in the non-irradiated group, while the irradiated group was entirely caused by malignancy (100%). As radiotherapy and chemotherapy are essential for middle- and late-stage malignancies, they were performed significantly more frequently in the irradiated group than in the non-irradiated group.

**Table 1:** Patient demographics and characteristics.

Characteristic	Total *n* = 64	Non-irradiated group (*n* = 20)	Irradiated group (*n* = 44)	*p*-value
Age, y (range)	51.9 ± 12.6	46.3 ± 12.4	54.5 ± 12.0*	0.015
Age on diagnosis, y (range)	48.2 ± 11.5	43.8 ± 12.1	50.1 ± 10.8*	0.04
BMI (range)	21.3 ± 2.5	21.3 ± 2.6	21.3 ± 2.5	0.948
Smoking (*n*, %)	1 (1.5%)	1 (5%)	0 (0%)	0.312
Comorbidity (*n*, %)				
Diabetes	7 (10.9%)	1 (5%)	6 (13.6%)	0.419
Hypertension	20 (31.3%)	4 (20%)	16 (36.4%)	0.251
Heart disease	9 (14.6%)	1 (5%)	8 (18.2%)	0.252
Etiology (*n*, %)				
Malignancy	46 (71.9%)	2 (10%)	44 (100%)*	<0.01
Benign gynecologic surgery	17 (26.6%)	17 (85%)	0 (0%)*	<0.01
Obstetric	1 (1.5%)	1 (5%)	0 (0%)	0.312
Radiotherapy (*n*, %)	44 (68.8%)	0 (0%)	44 (100%)*	<0.01
Chemotherapy (*n*, %)	25 (39.1%)	0 (0%)	25 (56.8%)*	<0.01

*p < 0.05, non-irradiated vs. irradiated.

Clinical data including fistula site, fistula size [according to the Goh’s classification (11)], vaginal width, other fistula and history of previous fistula repair were collected as pre-operation evaluation, which were shown in Table 2. Most VVFs were located at the trigonal site in both the non-irradiated (70%) and irradiated groups (61.4%). The fistula sizes were significantly different between the two groups. In the non-irradiated group, nearly half of the fistulas were smaller than 1.5 cm (55%) and barely larger than 3 cm (10%), whereas those were mostly larger than 1.5 cm (45.4% medium and 36.4% large) in the irradiated group. The irradiated group was further divided into three subgroups, among which the fistula sizes were smaller than 3 cm (76.2% small and 23.8% medium), >3 cm (100%) and >1.5 cm (70% medium and 30% large) respectively. Transvaginal fistula repair requires sufficient vaginal space for fistula exposition and surgical procedures. Thus, we have listed vaginal width as an important factor. In the irradiated group, the vaginal widths were significantly smaller than those in the non-irradiated group (*p* < 0.05). Regarding subgroup analysis, the vaginal widths were all > 3 cm in the fistula repair group and <3 cm in the urinary diversion group. Next, patients were found to have more other fistulas simultaneously in the irradiated group, especially rectovaginal fistulas (25%, *p* < 0.05). Both groups had a similar rate of previous fistula repair (25% vs. 20.5%).

**Table 2:** Pre-operation evaluation.

Variable		Non-irradiated group (*n* = 20)	Irradiated group (*n* = 44)			
			Total	Fistula repair (*n* = 21)	Urinary diversion (*n* = 13)	Fistula repair and urinary diversion (*n* = 10)
Fistula site (*n*, %)						
Trigonal		14 (70%)	27 (61.4%)	17 (81.0%)	3 (23.1%)	7 (70.0%)
Supratrigonal		6 (30%)	17 (38.6%)	4 (19.0%)	10 (76.9%)	3 (30.0%)
Fistula size (Goh’s classification) (*n*, %)						
<1.5 cm (small)		11 (55%)	8 (18.2%)	16 (76.2%)	0 (0%)	0 (0%)
1.5-3 cm (medium)		7 (35%)	20 (45.4%)	5 (23.8%)	0 (0%)	7 (70.0%)
>3 cm (large)		2 (10%)	16 (36.4%)*	0 (0%)	13 (100%)	3 (30.0%)
Vaginal width (*n*, %)						
<3 cm		1 (5%)	15 (34.1%)*	0 (0%)	13 (100%)	2 (20.0%)
> = 3 cm		19 (95%)	29 (65.9%)*	21 (100%)	0 (0%)	8 (80.0%)
Other fistula (*n*, %)						
Vesicorectal fistula		0 (0%)	5 (11.3%)	0 (0%)	4 (30.8%)	1 (10.0%)
Rectovaginal fistula		0 (0%)	11 (25.0%)*	1 (4.8%)	8 (61.5%)	2 (20.0%)
Previous fistula repair (*n*, %)		5 (25%)	9 (20.5%)	5 (23.8%)	1 (7.7%)	3 (30.0%)

*p < 0.05, non-irradiated vs. irradiated.

We performed different surgical treatments in each group, as shown in Table 3. For the non-irradiated group, we performed fistula repair surgery in all patient (20/20, 100%), while only 47.7% (21/44) in the irradiated group underwent fistula repair alone, with the remaining undergoing urinary diversion alone (13/44, 29.5%) or fistula repair + urinary diversion (10/44, 22.7%). Most fistula repair surgeries were performed using the transvaginal approach in both groups. On the other hand, most fistula repair surgeries were open surgeries in both groups. Tissue interposition was more frequently used in the irradiated group (20% vs. 58.1%). For the irradiated group, the gracilis muscle flap was most frequently used for tissue interposition (25.8%, *p* < 0.05). The irradiated group had a higher probability of urinary diversion, whereas no urinary diversion was performed in the non-irradiated group. Cystostomy was the most common procedure in the irradiated group (43.8%).

**Table 3:** Surgical procedure.

Procedure	Non-irradiated group (*n* = 20)	Irradiated group (*n* = 44)			
		Total	Fistula repair (*n* = 21)	Urinary diversion (*n* = 13)	Fistula repair and urinary diversion (*n* = 10)
Fistula repair only (*n*, %)	20 (100%)	21 (47.7%)*	21 (100%)	0 (0%)	0 (0%)
Urinary diversion only (*n*, %)	0 (0%)	13 (29.5%)*	0 (0%)	13 (100%)	0 (0%)
Fistula repair and urinary diversion (*n*, %)	0 (0%)	10 (22.7%)*	0 (0%)	0 (0%)	10 (100%)
Surgical approach of fistula repair (*n*, %)					
Transvaginal	16/20 (80%)	27/31 (87.1%)	17 (81.0%)	0 (0%)	10 (100%)
Transabdominal	4/20 (20%)	4/31 (12.9%)	4 (19.0%)	0 (0%)	0 (0%)
Type of surgery of fistula repair (*n*, %)					
Open	17/20 (85%)	30/31 (96.8%)	20 (95.2%)	0 (0%)	10 (100%)
Laparoscopic	2/20 (10%)	1/31 (3.2%)	1 (4.8%)	0 (0%)	0 (0%)
Robotic	1/20 (5%)	0/31 (0%)	0 (0%)	0 (0%)	0 (0%)
Tissue interposition (*n*, %)					
Martius flap	1/20 (5%)	2/31 (6.5%)	1 (4.8%)	0 (0%)	1 (10%)
Labial skin flap	1/20 (5%)	5/31 (16.1%)	3 (14.3%)	0 (0%)	2 (20%)
Omentum	1/20 (5%)	3/31 (9.7%)	3 (14.3%)	0 (0%)	0 (0%)
Gracilis muscle flap	1/20 (5%)	8/31 (25.8%) *	1 (4.8%)	0 (0%)	7 (70%)
None	16/20 (80%)	13/31 (41.9%)	13 (61.9%)	0 (0%)	0 (0%)
Urinary diversion (*n*, %)					
Nephrostomy	0/0 (0%)	2/23 (8.7%)	0 (0%)	2 (15.4%)	0 (0%)
Cutaneous Ureterostomy	0/0 (0%)	6/23 (26.1%)	0 (0%)	6 (46.2%)	0 (0%)
Cystostomy	0/0 (0%)	10/23 (43.8%) *	0 (0%)	0 (0%)	10 (100%)
Ileal conduit	0/0 (0%)	3/23 (13.0%)	0 (0%)	3 (23.1%)	0 (0%)
Augmentation cystoplasty	0/0 (0%)	2/23 (8.7%)	0 (0%)	2 (15.4%)	0 (0%)

*p < 0.05, non-irradiated vs. irradiated.

The surgical outcomes are presented in Table 4. In terms of follow-up time, there was no significant difference between the non-irradiated and irradiated groups (61.5[45–89] vs. 44.5[21.25–74.75]). The total operation time was also not different between the two groups (128.9 ± 47.0 vs. 146.4 ± 47.3). However, the fistula repair + urinary diversion subgroup had the longest total operation time compared with the other two subgroups (*p* < 0.05). The irradiated group exhibited significantly greater blood loss than the non-irradiated group (*p* < 0.05). There were no significant differences among the three subgroups. The number of postoperative hospital stay days did not differ between the non-irradiated and irradiated groups. However, the urinary diversion and the fistula repair + urinary diversion subgroups had significantly longer hospital stays than the fistula repair subgroup. The irradiated group had higher fistula recurrence rate than the non-irradiated group in both transvaginal approach (5/27, 18.5% vs. 1/16, 6.25%) and transabdominal approach (2/4, 50% vs. 0/4, 0%). The reoperation rate was 5% in the non-irradiated group and 11.4% in the irradiated group, with no significant difference.

**Table 4:** Surgical outcomes.

Variable	Non-irradiated group (*n* = 20)	Irradiated group (*n* = 44)			
		Total	Fistula repair (*n* = 21)	Urinary diversion (*n* = 13)	Fistula repair and urinary diversion (*n* = 10)
Follow up (month)	61.5 (45.0, 89.0)	44.5 (21.25, 74.75)	43.00 (15.00, 73.00)	51.0 (33.0, 90.0)	45.5 (26.75, 57.0)
Total operation time (min)	128.9 ± 47.0	146.4 ± 47.3	137.0 ± 26.3	128.8 ± 66.4	189.1 ± 24.2**
Blood loss (ml)	20.5 (14.75, 40.5)	46.5 (21.5, 80.5)*	45.0 (25.0, 57.0)	22.0 (17.0, 194.0)	81.0 (49.25, 108.25)
Post-operative hospital stay (day)	4.0 (3.0, 6.0)	5.0 (4.0, 6.0)	4.0 (3.0, 5.0)	6.0 (5.0, 11.0)***	5.5 (5.0, 6.75)****
Fistula recurrence (*n*, %)					
Transvaginal	1/16 (6.25%)	5/27 (18.5%)	5/17 (29.4%)	0/13 (0%)	0/10 (0%)
Transabdominal	0/4 (0%)	2/4 (50%)	2/4 (50%)	0/13 (0%)	0/10 (0%)
Reoperation (*n*, %)	1/20 (5%)	5/44 (11.4%)	5/21(23.8%)	0/13 (0%)	0/10 (10%)

Mean (10%) and deviation.

Median, (IQR).

*p < 0.05, non-irradiated vs. irradiated.

**p < 0.05, FR vs. FR+UD, and UD vs. FR+UD.

***p < 0.05, FR vs. UD.

****p < 0.05, FR vs. FR+UD.

We further analyze the outcomes of patients had previous repair (redo patients) since it is clinically critical in the comprehensive management and prognostication of VVF. In the non-irradiated group, there were 5 patients and the fistulas all exceed 1.5 cm. We performed fistula repair, mostly with tissue interposition and achieved an 80% (4/5) success rate, which was moderately lower than the primary surgery patients (100%, 15/15) in the same group. As for the irradiated group, there were 9 patients, of whom the fistulas exceed 1.5 cm and the vaginal width less than 3 cm. Among them, 5 cases underwent fistula repair combined with tissue interposition, 1 underwent urinary diversion, and 3 underwent fistula repair combined with urinary diversion. Notably, the success rate of the first subgroup was only 40% (2/5), which was significantly lower than the primary surgery patients (75%, 12/16) in the irradiated group. These findings demonstrate a significantly lower success rate for secondary fistula repair in patients with prior radiotherapy, likely attributable to compromised local tissue conditions, such as fibrosis and poor vascularity, as well as increased technical difficulty of surgical procedures.

Postoperative complications are presented in Table 5. A total of 30 complications occurred in the irradiated group and only 8 in the non-irradiated group. The top two early complications were the same in both groups: urinary tract infection and bladder spasms. All other early complications occurred in the irradiated group rather than in the non-irradiated group. The fistula repair subgroup and the fistula repair + urinary diversion subgroup had similar complications in terms of both proportion and type. Only Grade I complications were recorded in both groups except from the diversion subgroup. Grade II and III complications (anastomotic leak, postoperative hemorrhage, and postoperative ileus) were caused by the procedures related to intestine in the diversion surgery. Regarding the late complications, 1 was observed in the non-irradiated group while 8 were in the irradiated group. The ratios are relatively low (overactive bladder 11.4%, underactive bladder 2.3%, chronic pelvic pain 4.5%), and these symptoms could be alleviated by medication or conservative treatments.

**Table 5:** Complications.

Complication type	Non-irradiated group (*n* = 20)	Irradiated group (*n* = 44)			
		Total	Fistula repair (*n* = 21)	Urinary diversion (*n* = 13)	Fistula repair and urinary diversion (*n* = 10)
Early complications	7 (35%)	22 (50%)	11 (52.4%)	7 (53.8%)	4 (40%)
Urinary tract infection (*n*)	5 (25%)	9 (20.5%)	5 (23.8%)	2 (15.4%)	2 (20%)
Surgical site infection (*n*)	0 (0%)	2 (4.5%)	0 (0%)	1 (7.7%)	1 (10%)
Bladder spasms (*n*)	2 (10%)	8 (18.2%)	6 (28.6%)	1 (7.7%)	1 (10%)
Anastomotic leak (*n*)	0 (0%)	1 (2.3%)	0 (0%)	1 (7.7%)	0 (0%)
Postoperative hemorrhage (*n*)	0 (0%)	1 (2.3%)	0 (0%)	1 (7.7%)	0 (0%)
Postoperative ileus (*n*)	0 (0%)	1 (2.3%)	0 (0%)	1 (7.7%)	0 (0%)
Clavien-Dindo classification					
Grade I	7	19	11	4	4
Grade II	0	2	0	2	0
Grade III	0	1	0	1	0
Late complications	1 (5%)	8 (18.2)	5 (23.8%)	1 (7.7%)	2 (20%)
Overactive bladder (*n*)	1 (5%)	5 (11.4%)	3 (14.3%)	0 (0%)	2 (20%)
Underactive bladder (*n*)	0 (0%)	1 (2.3%)	1 (4.8%)	0 (0%)	0 (0%)
Chronic pelvic pain (*n*)	0 (0%)	2 (4.5%)	1 (4.8%)	1 (7.7%)	0 (0%)

## Discussion

The VVF is a disastrous condition that affects women physically, psychologically, emotionally, and economically (1, 12). Successful VVF repair significantly improves the quality of life and recovers patients’ self-esteem (13). Although the literature is robust with different approaches to VVF repair, none is universally acknowledged to be the best (14). Meanwhile, situations such as extensive tissue defects, narrow vagina, and frozen pelvic (mainly caused by radiation) may render a fistula “inoperable” (15). Urinary diversion appears to be the best option for those VVF patients with “inoperable” fistula. Unfortunately, a standard algorithm for decision-making regarding the type and approach of surgical treatment of VVFs has not yet been reported.

In the current study, we reviewed 64 patients with VVF who underwent surgical treatment at our center during a 12-year period. We found that the patients’ demographics, fistula characteristics, surgical treatments, and outcomes differed significantly between the non-irradiated and irradiated groups. Our study showed that the patients in non-irradiated group were younger at age of diagnosis and surgery, and the etiology was benign gynecologic surgery rather than malignancy for those in the irradiated group. Regarding fistula characteristics, our study demonstrated that the fistulas were smaller, the vagina was more flexible, and there were fewer other fistulas in the non-irradiated group. In terms of surgical treatments and outcomes, direct fistula repair was successful in most non-irradiated patients, whereas different methods were used in irradiated patients, with a relatively lower success rate and more complications.

The final surgical treatments (fistula repair or urinary diversion) were determined by the feasibility and success probability of fistula repair, which are affected by multiple factors, such as fistula size and peri-fistula tissue condition. Previous studies have classified fistulas into simple and complex types. Simple fistulas manifest as isolated, non-irradiated fistulas that are small in size (<0.5 cm). Complex fistulas include cases with size ≥ 2.5 cm, multiple fistulas, previous history of failed fistula repair, postradiation-induced fistulas, or those associated with malignancy. In our experience, fistulas with a size ≤3 cm can mostly undergo repair surgery, and the success rate is acceptably high in both non-irradiated and irradiated patients. When the size was > 3 cm, direct fistula closure was still possible in the non-irradiated group, whereas urinary diversion was the first option in the irradiated group.

Common surgical repair approaches for VVF include transvaginal and transabdominal approaches. In most cases, the transvaginal approach is feasible (12) and widely preferred because of the advantages of minimal surgical injury, less bleeding, and short durations of operation and hospital stay (16). However, the transvaginal approach is difficult when the vaginal exposure is inadequate, and the fistulas are highly supratrigonal or close to the ureteric ostium (17). In these situations, the transabdominal approach is recommended. In our study, we also prioritized the transvaginal approach. We considered vaginal width as an indicator for adequate vaginal exposure. When the width is smaller than 3 cm, fistula repair is difficult to achieve, and urinary diversion should be chosen first. Interestingly, we found that the fistula recurrence rate of the transabdominal approach was similar to that of the transvaginal approach in the non-irradiated group, while it was significantly higher than that in the irradiated group. This contradictory phenomenon may be due to the side effects of radiation: (1) pelvic organ/tissue adhesion, which results in more difficult surgical procedures, such as separating the bladder and vagina; (2) less pelvic blood supply and tissue necrosis, which leads to impaired wound healing, causing failure of repair surgery.

With the development of minimally invasive techniques, multiple novel approaches have been reported for VVF repair from laparoscopic to robotic. Generally, the minimal invasive surgery was performed by a transabdominal approach, which can lead to reduced blood loss, decreased morbidity, shorter hospital stay and faster recovery compared to the traditional open transabdominal approach (3). Meanwhile, with the popularity of single port equipment, minimal invasive surgery, especially robotics, has garnered more attention in transvaginal VVF repair. Differ to routine open transvaginal repair, the robotic transvaginal approach can be accomplished for high position VVF or limited vaginal space, where traditional surgery may struggle to adequately expose the fistula and demands exceptionally high surgical skills (18). In our study, we mainly utilized open approach for both transvaginal and transabdominal repair. For the non-irradiated group, we performed three laparoscopic transabdominal surgery and one robotic transvaginal surgery and all get successful repair. However, for the radiation group, the severe pelvic adhesion and tissue scar hampered the docking and separation procedures. We performed only one case though achieved a good result. We recommend the single-port robotic transvaginal repair for suitable cases (high-position, plenty vaginal space), because it has the advantage of both transvaginal and minimally invasive surgery, leading to less trauma, faster recovery and high successful rate.

Generally, simple VVF can be fixed by a layer-by-layer surgical suturing of the vaginal and bladder wall and get a high successful rate. While complex and recurrent fistulas should be treated with the interposition of tissue grafts (19). Tissue grafts improve local neovascularization, absorb urinary extravasate, prevent leakage of urine from the bladder and promote wound healing (20). Various tissue grafts can be used and the choice depends on surgical approach. Omentum, peritoneum and pedicled rectus abdominis muscle flaps can be utilized during transabdominal approaches. Labial fat tissue flap (also known as Martius flap), labial skin flap, and gracilis muscle flap can be employed transvaginally. In our cohort, we performed fistula repair with tissue interposition in four cases of the non-irradiated group (4/20). Three cases were via transvaginal approach (one Martius flap, one labial skin flap, and one gracilis muscle flap) and only one was failed. One case was through transabdominal approach by omentum without fistula recurrence. In the irradiated group, 18 patients received simultaneous tissue interposition when performing fistula repair (18/31) with or without urinary diversion (8/21 vs. 10/10). The tissue interposition indeed supported a relatively high successful rate of fistula repair. Accordingly, we suggested that fistula repair with tissue interposition in a transvaginal approach is a promising method in the radiation-related VVFs.

Radiation can cause extensive tissue destruction, narrow vagina, frozen pelvic, results in “inoperable” fistulae. Permanent urinary diversion, therefore, appears to be the final option for VVF patients with inoperable fistulae (21). However, permanent urinary diversion is not to be taken lightly. It is important that patients fully understood the risks and rationale for the surgery before making the decision. There are various methods for urinary diversion including nephrostomy, cutaneous ureterostomy, cystostomy, ileal conduit, and augmentation cystoplasty, with different advantages and disadvantages. In our study, cutaneous ureterostomy is the first choice for the elder patients since it can completely solve the incontinence, achieve a high quality of life and is easy to perform with less complications. Nephrostomy is considered for those patients are not bearable for general anesthesia. Ileal conduit and augmentation cystoplasty are recommended for young patients persuing high quality of life though the surgery is difficult and can cause severe complications such as postoperative hemorrhage, ileus, even death. What needs to be mentioned is that we perform simultaneous permanent urinary diversion (cystostomy) and fistula repair in 10 radiation-related VVF patients and get a 100% successful rate and 0% fistula recurrence. The result showed that permanent cystostomy can significantly enhance the successful rate of fistula repair, which should be good option for complex radiation-related VVF patients. Radiation-induced cystitis can cause the bladder dysfunction and tissue damage, which results in disability of wound healing and relapse of fistula. The possible mechanism of permanent cystostomy may be that it creates an optimal healing environment by eliminating urine irritation, maintaining low intravesical pressure, and protecting the repair from mechanical disruption during bladder filling.

**Figure 2:**
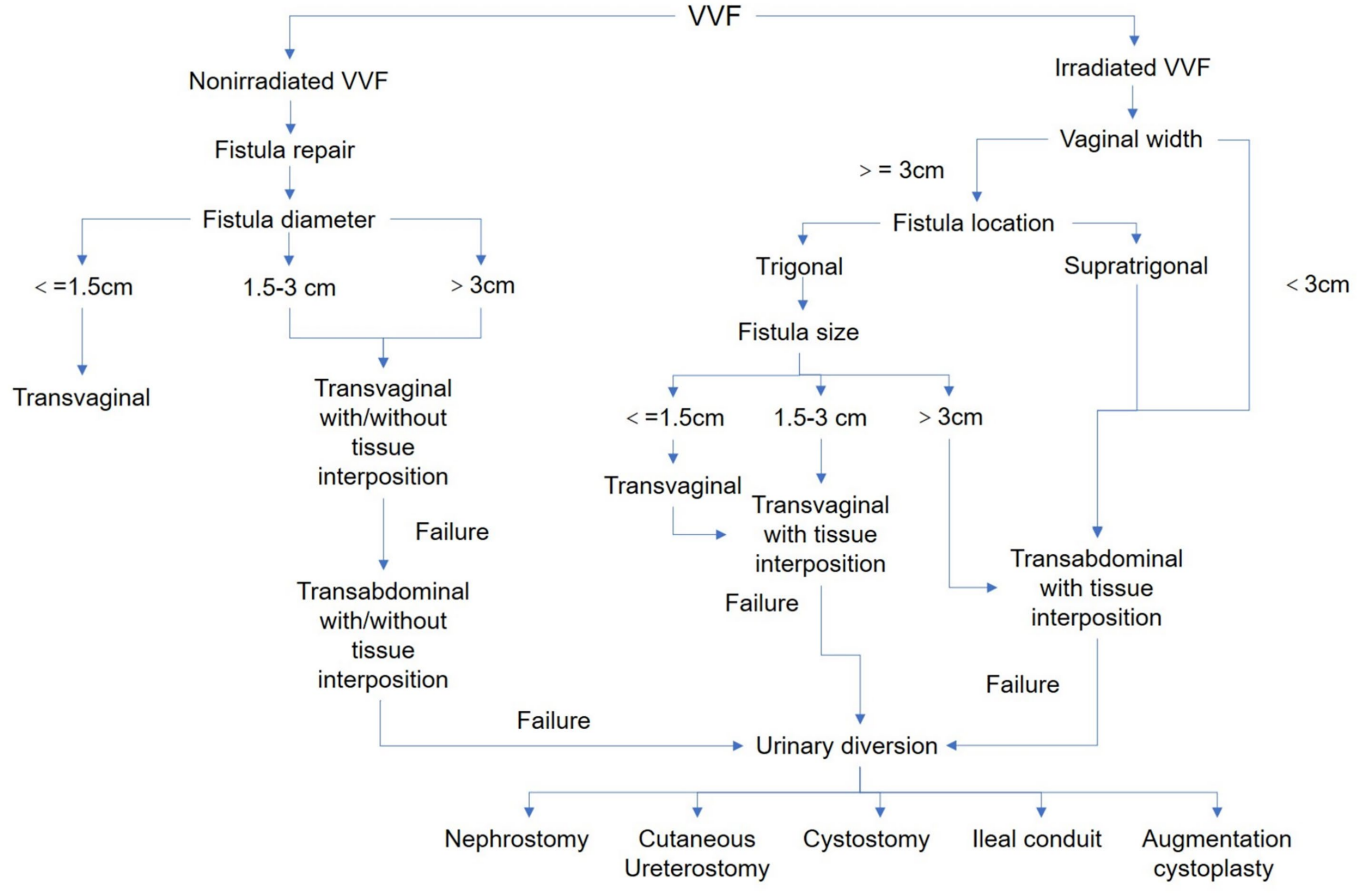
Algorithm for the surgical management of VVF.

Based on the data of our retrospective study, as well as the previously published literature, we established an algorithm detailing treatment in patients with VVFs (Figure 2). The major branch point of the algorithm is history of pelvic radiation, fistula size, vaginal width and fistula location. Since radiation can lead to significant tissue damage, increase the difficulty of surgery and decrease success rate of fistula rate, we put it as the first decision node. For non-irradiated VVF, we achieved a 93.75% success rate of fistula repair by transvaginal approach and recommend it as the first option, followed by tissue interposition and transabdominal approach. We set the vaginal width as a second node because it changes the surgical method totally. The vaginal width is almost larger than 3 m without radiation, which is suitable for transvaginal procedures. However, radiation can cause significant vaginal tissue fibrosis, and often leads to frozen pelvic and small vaginal width. When it is smaller than 3 cm, the vagina tissue is usually rigid for well-exposure and there is extremely limited space for the surgical procedure which makes transvaginal approach and direct fistula repair nearly impossible mission. The next node is fistula size, which can be clearly classified as Goh’s classification. It is commonly recognized that the larger the fistula is, the harder it can be repaired. From out data, fistula smaller than 1.5 cm has a high success rate of direct repair. Tissue interposition is suggested to increase the success rate when the size is between 1.5 and 3 cm. When it is larger than 3 cm, the success rate of repair is significantly low. The final node is the fistula location, which can be quickly remembered that trigonal for transvaginal while supratrigonal for transabdominal. Specifically, for radiation VVF, if the vaginal width is larger than 3 cm, fistula is located at trigonum, fistula size is smaller than 3 cm, fistula repair with or without tissue interposition by transvaginal approach is also the first choice. When the vaginal width is smaller than 3 cm, the fistula is located at supratrigonal region, or the fistula size is larger than 3 cm, fistula repair with or without tissue interposition by transvaginal approach is preferred. The final option is urinary diversion for all circumstances.

This study has some limitations. First, this study is limited by its retrospective nature and single-center design with potential selection bias. Second, the sample size is relatively small, especially when divided into subgroups, which limits the statistical power of analysis and the final conclusion. Third, the study lacks standardized, validated quality-of-life (QoL) measures to assess true “success” from the patient’s perspective. Without incorporating patient-reported outcome measures, the definition of “success” remains incomplete and surgeon-centered rather than patient-centered. Lastly, the algorithm is derived only from our limited experience and needs further validation. These limitations underscore the need for larger, prospective, multi-center investigations that incorporate patient-centered outcomes to validate and refine the proposed treatment algorithm.

## Conclusion

Based on our experience, we proposed a management algorithm for surgical management for both irradiated and non-irradiated VVFs. Transvaginal approach is the first option for fistula repair due to its minimal invasion and high success rate. Transvaginal fistula repair with tissue interposition combined with permanent cystostomy also demonstrated high success for irradiated VVFs in our series which can both treat incontinence and maintain a high quality of life. Further studies including more VVF patients are needed to verify the algorithm and optimize the details.

## Data Availability

The original contributions presented in the study are included in the article/supplementary material, further inquiries can be directed to the corresponding authors.
